# Optimal Sequence for Total Neoadjuvant Therapy in Locally Advanced Rectal Cancer: An Evidence‐Based Review

**DOI:** 10.1002/cam4.70291

**Published:** 2024-10-10

**Authors:** Reza Ghalehtaki, Forouzan Nourbakhsh, Romina Abyaneh, Azadeh Sharifian, Sheyda Pashapour‐Khoyi, Mahdi Aghili, Maria Antonietta Gambacorta, Felipe Couñago

**Affiliations:** ^1^ Department of Radiation Oncology Cancer Institute, IKHC, School of Medicine, Tehran University of Medical Sciences Tehran Iran; ^2^ Radiation Oncology Research Center Cancer Research Institute, IKHC, Tehran University of Medical Sciences Tehran Iran; ^3^ UOC di Radioterapia Oncologica, Dipartimento di Diagnostica per Immagini Radioterapia Oncologica Ed Ematologia, Fondazione Policlinico Universitario A. Gemelli IRCCS Rome Italy; ^4^ Department of Radiation Oncology GenesisCare, Hospital Universitario Vithas Madrid La Milagrosa Madrid Spain

**Keywords:** adenocarcinoma, chemotherapy, radiotherapy, rectal cancer, total neoadjuvant therapy

## Abstract

**Introduction:**

Historically, multimodal therapeutic strategies involving preoperative chemoradiotherapy (CRT), surgery, and adjuvant chemotherapy (CT) have been employed to treat locally advanced rectal cancer (LARC). Total Neoadjuvant Therapy (TNT) is showing promise in improving outcomes. Despite its benefits, the optimal sequencing within TNT—whether induction chemotherapy should precede or follow chemoradiotherapy—remains a critical question. This study endeavors to explore the effects of different TNT sequencing strategies on patient outcomes, including tumor downstaging, pathological response, organ preservation, and the balance between efficacy and tolerability.

**Methods:**

Our methodology entailed a comprehensive literature review conducted on Medline, focusing on recent research, including retrospective studies, systematic reviews, and clinical trials. The review emphasized the comparison of induction chemotherapy versus consolidation chemotherapy within TNT regimens, assessing outcomes such as pathological response, organ preservation rates, and adverse effects. To ensure the selection of appropriate and high‐quality studies, specific inclusion and exclusion criteria were applied.

**Results:**

The analysis revealed that induction chemotherapy might lead to decreased adherence to subsequent chemoradiotherapy while offering an early intervention against micrometastasis and potentially improving overall chemotherapy compliance. Conversely, consolidation chemotherapy has been associated with higher pathological complete response (pCR) rates and improved tolerability, indicating its potential for patients requiring local symptom relief or those eligible for a nonoperative management approach. Comparative studies like CAO/ARO/AIO‐12 and the OPRA trials have significantly contributed to our understanding, suggesting that while both strategies have distinct advantages, the choice between induction and consolidation chemotherapy should be tailored based on individual patient profiles and tumor characteristics.

**Conclusion:**

This narrative review underscores the importance of a nuanced approach to TNT sequencing in locally advanced rectal cancer, highlighting the need for further research to refine treatment strategies.

## 1 Introduction

Historically, locally advanced rectal cancer (LARC) has been treated employing multimodal therapeutic strategies, including neoadjuvant chemoradiotherapy (nCRT), surgery, and adjuvant chemotherapy (CT). This approach has been traditionally adopted to improve patients' long‐term clinical outcomes and survival rates. Neoadjuvant CRT is administered to reduce tumor size, enhance surgical resectability, and enhance local control. Surgery is then performed to remove the tumor, and adjuvant CT is given to prevent recurrence and improve survival rates. Although multimodal treatment strategies have been effective in the management of LARC, there is a growing need to explore alternative or complementary therapeutic approaches to optimize clinical outcomes and patient quality of life [1].

After advancements in radiotherapy and surgery, the local recurrence rate in LARC has diminished to as few as 2%–5%. However, distant metastasis still remains a leading cause of treatment failure, occurring in approximately 25% of cases [2]. As a result, systemic chemotherapy has become a key component in multimodal therapy, and improving patient compliance to better tolerate chemotherapy is crucial. Unfortunately, the suboptimal compliance of patients to receive planned cycles of adjuvant chemotherapy completely after surgery is a common challenge that can greatly impact patient outcomes and quality of life [3]. To address this issue, total neoadjuvant therapy (TNT) was developed as a new approach to improve the outcomes of LARC treatment. TNT is a comprehensive presurgical treatment plan that aims to enhance patient compliance and impact micrometastasis. Evidence has shown that TNT results in higher rates of complete pathological response and significantly improves disease‐free and overall survival without a significant increase in severe adverse effects [4, 5].

Based on large Phase III randomized clinical trials, two popular TNT schedules include either induction chemotherapy followed by chemoradiotherapy (PRODIGE‐23‐like approach) or radiotherapy followed by consolidation chemotherapy (RAPIDO‐like approach) [6, 7]. Both approaches have shown superior outcomes in comparison to conventional or standard long‐course chemoradiotherapy. Despite these promising outcomes, the optimal sequence of treatment modalities within TNT, specifically the choice between induction chemotherapy and consolidation chemotherapy, remains a crucial question. It is necessary to examine how the sequencing of treatment affects outcomes, adverse effects, and rates of organ preservation to reduce uncertainty and improve treatment efficacy.

This narrative review aimed to provide a comprehensive overview of the sequence of treatment modalities in TNT for LARC. Through a narrative review, we will explore the different sequencing strategies on patient outcomes, with a focus on tumor downstaging, pathological response, organ preservation, and the balance between efficacy and tolerability. By analyzing the nuances of TNT sequencing, this work aimed to contribute to the ongoing refinement of treatment paradigms and ultimately improve LARC management.

## 2 Methods

We extensively searched the Medline database for this review, covering literature published over the past 10 years. Our focus was on identifying studies related to rectal cancer and total neoadjuvant treatment, specifically investigating the use of induction and consolidation chemotherapy in patients with locally advanced rectal cancer. We included a range of study designs, such as retrospective studies and prospective clinical trials, to ensure a comprehensive understanding of TNT. The primary outcomes of interest were pathological response rates, organ preservation outcomes, and adverse effects associated with various TNT regimens. We applied rigorous inclusion criteria, selecting studies that focused on adult patients with LARC, clearly defined the TNT approach (induction or consolidation), and provided detailed outcome data. Exclusion criteria were applied to studies with poorly defined treatment protocols, insufficient outcome data, or irrelevant patient populations. This approach allowed us to evaluate and compare the effectiveness and safety profiles of induction versus consolidation chemotherapy and overall trends and findings across different TNT strategies in LARC management.

### 2.1 Induction Chemotherapy Studies

Since the major cause of treatment failure in LARC is distant metastasis, some investigators suggested that by starting the treatment with chemotherapy, the micrometastasis can be addressed earlier so the outcomes would be better. Induction chemotherapy (IC) may also be used to decrease the primary tumor size and relieve associated symptoms like rectal bleeding, obstruction, and pain without requiring immediate radiotherapy. However, some argue that IC may increase toxicity and decrease patient compliance with subsequent radiation therapy, which is considered the more effective component of treatment [4, 8, 9, 10]. Below, we review the studies that addressed the efficacy and safety of IC as part of a TNT protocol.

In the GCR‐3 trial, researchers investigated the effectiveness of induction chemotherapy (IC) in 108 patients with LARC. The participants were randomly allocated to two groups: Arm A was given neoadjuvant CRT with capecitabine and oxaliplatin, followed by resection and adjuvant chemotherapy; Arm B underwent IC for 4 cycles, followed by CRT and resection. The study's main outcome was pathological complete response (pCR), which showed that the two groups did not display any notable difference (13.5% in Arm A vs. 14.3% in Arm B, *p* = 0.9). Additionally, both groups showed similar rates of tumor downstaging and R0 resection. However, the IC group had better compliance and fewer side effects [11]. The long‐term update also showed similar OS, DFS, and incidence of LR and DM [12]. The important point is that the rate of chemotherapy initiation and completion was much higher in this study compared with the landmark German Rectal Cancer Study (96% vs. 71% for initiation and 93% vs. 54% for completion) [13].

In the EudraCT trial, two different treatment regimens were evaluated in 57 patients who had T2‐4 rectal cancer and/or lymph node involvement. Arm A, which consisted of 29 patients, received 25 fractions, adding up to a total of 45 Gy using 3D‐conformal radiotherapy plus simultaneous 5‐FU, followed by surgery. On the other hand, Arm B (28 patients) underwent two cycles of mFOLFOX6, followed by the same chemoradiotherapy regimen and then surgery, similar to the first group's postchemotherapy protocol. The study's main goal was to assess the ypT0‐1 response, and there was no notable difference between the two groups (34% in Arm A vs. 32% in Arm B, *p* = 0.85). Additionally, pathological complete response (pCR) rates were also found to be similar (28% in Arm A vs. 25% in Arm B, *p* = 0.92). The researchers did not observe a notable difference in sphincter preservation rates between the two arms. A higher total Grade 3–4 toxicity was reported in Arm B (36% vs. 8% in Arm A, *p* = 0.017) [14].

In 2018, Cercek and colleagues in MSKCC carried out a retrospective study on 628 individuals with LARC. The study compared two treatment methods: the standard arm (320 patients) received CRT followed by delayed surgery and adjuvant chemotherapy, while the IC arm (308 patients) received upfront chemotherapy followed by CRT, similar to the standard arm. The CRT comprised 45 Gy to the pelvis plus a 5.4 Gy boost, with concurrent 5‐FU or capecitabine. It is important to note that the IC arm had a higher complete response rate, including sustained clinical and pathological response, compared with the standard arm. The rate of complete response in the IC and standard arms was 36% vs. 21%, respectively. Of those who underwent surgery, the pCR rate in the IC and standard arms was 18% vs. 17%, respectively. Additionally, the sustained clinical complete response (cCR) rate in the IC and standard arms was 92% vs. 79%, respectively. The study also reported higher treatment compliance and local control in the IC arm, indicating the potential advantages of this treatment strategy for nonoperative management in selected patients [15].

In the Phase II GEMCAD‐1402 trial, 180 patients aged between 18 and 75 years, diagnosed via centrally reviewed MRI to have middle or distal T3c‐d/T4/N2, were recruited. Patients were randomized into the intervention arm to receive either 3 months of mFOLFOX6 plus aflibercept (4 mg/kg) or mFOLFOX6 alone, followed by CRT and TME in both arms. The primary endpoint was pCR. The investigators reported that although the addition of aflibercept increased the rate of pCR from 13.8% to 22.6% (*p* = 0.15), it was at the cost of a higher rate of grade 3/4 hypertension. The tolerability of both regimens was higher than 92%. The postop complications were the same between groups. At 3 years, the DFSand OS rates were 75.2% vs. 81.5% and 89.3% vs. 90.7% in aflibercept+mFOLFOX6 vs. mFOLFOX6 alone arms, respectively. The authors emphasized on the role of molecular classification in the benefit gained from the addition of aflibercept. The rate of pCR was notably greater in the epithelial subtype compared with the mesenchymal subtype. In conclusion, although the rate of pCR was increased by adding aflibercept, 3‐year DFS, and OS were not changed. We should notice that the rate of pCR in the mFOLOFX6 alone arm was not so higher than standard CRT alone [16, 17]. Although this study primarily examines the impact of adding aflibercept to induction chemotherapy within the TNT approach, it also provides valuable insights into potential enhancements in induction chemotherapy strategies as part of TNT sequencing.

Another retrospective study conducted in 2021 by El Husseini at the American University of Beirut compared the effectiveness of induction chemotherapy followed by radiotherapy and surgery with conventional chemoradiotherapy (CRT) in treating patients with LARC (T3‐4/T2N+). In the TNT group, 26 patients underwent six cycles of mFOLFOX followed by short‐course radiotherapy and TME within 16–18 weeks. The CRT group consisted of 55 patients with long‐course radiotherapy (45 Gy over 5 weeks and simultaneous capecitabine), followed by TME and postoperative chemotherapy. The research revealed that the TNT group had a noticeably greater pCR rate of 38.5% than that in the CRT population, 27.3%, after a median follow‐up of 22.7 months for the TNT group and 47.8 months for the CRT group. Moreover, the 2‐year disease‐free survival (DFS) rates were similar between the TNT (81%) and CRT groups (84%). The study concluded that the TNT approach improved chemotherapy compliance (40% in the CRT group vs. 84.5% in the TNT group received full‐dose chemotherapy), pCR rates, and tumor downstaging [18].

In the famous UNICANCER PRODIGE‐23 Phase III trial, published in 2021, Conroy and colleagues compared induction chemotherapy followed by long‐course CRT (experimental arm) with neoadjuvant CRT alone (standard arm) in patients with LARC. The study enrolled 461 patients, with 231 receiving induction chemotherapy (mFOLFORINOX for 6 cycles), followed by CRT, TME, and adjuvant chemotherapy for 3 months, and 230 undergoing standard CRT, followed by surgery and adjuvant chemotherapy (FOLFOX/Capecitabine) for 6 months. The trial reported that the induction chemotherapy group had higher rates of pCR and better 3‐year DFS and metastasis‐free survival compared with the CRT group. However, the TNT group had more grade 3–4 adverse effects, such as neutropenia and diarrhea during chemoradiation therapy. While adjuvant chemotherapy is not considered a part of the TNT protocol, both treatment groups received the same adjuvant chemotherapy regimen with a longer duration in the CRT group. During the adjuvant chemotherapy period, lymphopenia was observed in 11.2% of patients in the TNT group vs. 27.1% in the CRT group. In terms of all toxicities, the standard CRT group experienced more grade 3/4 events than the TNT group (74.1% vs. 45.1%, *p* < 0.0001) [6]. The shorter duration of adjuvant chemotherapy in the TNT group likely reduced the cumulative toxic effects, potentially explaining why the TNT group had a better overall toxicity profile. Moreover, the superior outcomes observed in the TNT group, such as higher rates of pCR and better 3‐year DFS and metastasis‐free survival, indicate that the initial induction chemotherapy followed by CRT might be more effective in controlling the disease, despite a shorter course of adjuvant therapy. This suggests that the intensified treatment upfront in the TNT group could potentially allow for a reduction in the need for prolonged adjuvant therapy without compromising efficacy, thereby minimizing the risk of severe toxicities associated with extended chemotherapy.

**FIGURE 1:**
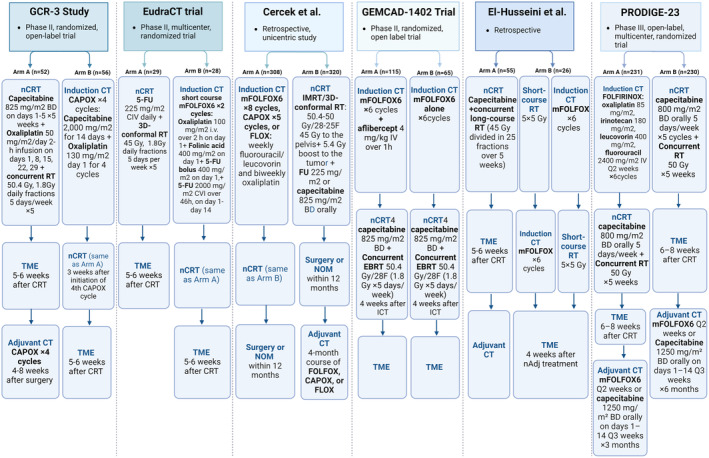
Design of studies evaluating induction chemotherapy as a TNT approach. This figure provides an overview of six different studies, including clinical trials and retrospective studies, that evaluated the use of induction chemotherapy before neoadjuvant chemoradiotherapy as part of the TNT approach in locally advanced rectal cancer treatment. Each column outlines a different study's treatment regimen, including the chemotherapy protocols, radiation therapy, and timing of surgery. CAPOX, Capecitabine, 1000 mg oral BD Days 1–14 + oxaliplatin 130 mg/m2 Day 1, Q3 weeks; CT, chemotherapy; EBRT, external beam radiation therapy; FOLFOX, Oxaliplatin 85 mg/m2 + 5‐FU CIV infusion 2400 mg/m2 days 1–3 ± 5‐FU 400 mg/m2 IV bolus + Leucovorin 400 mg/m2 Q2 weeks; mFOLFOX6, Oxaliplatin 85 mg/m^2^ 2h IV + Leucovorin 400 mg/m^2^ 2h IV + Fluorouracil 400 mg/m^2^ IV then 2400 mg/m^2^ CIV over 46 h Q2 weeks.nCRT, neoadjuvant chemoradiotherapy; NOM, nonoperative management; RT, radiation therapy; TME, total mesorectal excision.

Induction chemotherapy is a useful approach for LARC patients undergoing TNT regimens. Studies suggest that this method yields a higher rate of pCR compared with conventional treatments and is also associated with an overall favorable toxicity profile during the induction phase. Patients who receive induction chemotherapy are more likely to adhere to the complete chemotherapy regimen but may demonstrate decreased compliance with subsequent chemoradiotherapy. This underscores the importance of induction chemotherapy in customizing treatment plans based on the initial response of the patient and the expected tolerance to RT. The articles are summarized in Table 1, and study designs are illustrated in Figure 1.

**TABLE 1:** Results of studies evaluating induction chemotherapy.

Study name, type, and year of publication	Number of participants	pCR	R0 resection rate	Downstaging	Sphincter preservation	Toxicity	Treatment completion rate per protocol	OS	DFS	LR/DM	Median f/u
GCR‐3, Phase II RCT, 2010 (Fernandez‐Martos et al. 2010, 2015) [11, 12]	108 CRT *n* = 52, ICT *n* = 56	CRT: 13%, ICT: 14% (*p* = 0.94)	CRT: 87%, ICT: 86% (*p* = 0.40)	CRT: 58%, ICT: 43% (*p* = 0.13)	NR	Grade 3–4 toxicity during CT/RT CRT: 29%, ICT: 23% (*p* = 0.36) Grade 3–4 toxicity during adjuvant/induction CRT: 54%, ICT: 19% (*p* = 0.0004)	CRT: 54%, ICT: 91% (*p* = 0.0001), CRT group: Received adjCT: 76%, Received RT per protocol: CRT:80%, ICT: 85%	5‐year OS CRT: 78%, ICT: 75% (*p* = 0.64)	5‐year DFS CRT: 64%, ICT: 62% (*p* = 0.85)	5‐year LR incidence CRT: 2%, ICT: 5% (*p* = 0.64) 5‐year DM incidence CRT: 21%, ICT:23% (*p* = 0.79)	69.5 months
EudraCT, Phase II RCT, 2011 (Marechal et al. 2012) [14]	57 CRT *n* = 29, ICT *n* = 28	CRT: 28%, ICT: 25% (*p* = 0.92)	NR	CRT: 72%, ICT: 61% (*p* = 0.39)	Similar in both groups	Grade 3–4 toxicity during ICT: 21%, +1 death under ICT due to febrile neutropenia Preop CRT toxicity: no difference between the two groups (7%)	CRT arm: 97%, ICT arm:96% RT COMPLETION: CRT arm: 100%, ICT arm: 96%	NR	NR	NR	NR
Cercek et al. Retrospective unicentric, 2018 (Cercek et al. 2018) [15]	628 CRT *n* = 320, TNT *n* = 308	pCR: CRT:17%, TNT:18% CR (cCR + pCR): CRT: 21.3%, TNT: 35.7% 12‐months cCR in NOM CRT: 16%, TNT: 22% 24‐month cCR CRT:91%, TNT: 87%	NR	NR	NR	NR	**5‐FU**: Average % of the planned dose received CRT: 88.4%, TNT: 95.9 (*p* = 0.003) % of patients requiring dose reduction CRT: 50.5%, TNT: 25.7% (*p* < 0.001) **Oxaliplatin**: Average % of the planned dose received CRT:73.3%, TNT: 90.4% (*p* < 0.001) % of patients requiring dose reduction CRT: 76.2%, TNT: 53.8% (*p* < 0.001)	NR	NR	No patient with sustained cCR had a distant recurrence	CRT:23 months TNT: 40 months(*p* < 0.001)
GEMCAD‐1402, Phase II RCT, 2019 (Fernández‐Martos et al. 2019; Pesántez et al. 2023) [16, 17]	180 arm A (ICT + aflibercept) *n* = 115, arm B (w/o aflibercept) *n* = 65	Arm A: 22.6%, Arm B: 13.8% (*p* = 0.15)	Arm A: 98%, Arm B:96.7%	NR	LAR: ArmA: 74.7%, Arm B: 67.7%	Grade 3–4 hypertension was higher with the addition of aflibercept Grade 3/4 toxicity during ICT: Arm A: 51.3%, Arm B: 23% Grade 3/4 toxicity during CRT: Arm A: 17.1% Arm B: 7.8%	Received total dose of RT: Arm A: 92.3%, Arm B: 96.8% Received full dose of CT during RT: Arm A: 84.7% Arm B: 90.6%	3‐year OS: Arm A: 89.3%, Arm B: 90.7%	3‐year DFS: Arm B: 75.2%, Arm B: 81.5%	3‐year LR: Arm A: 5.2%, Arm B: 6.1% 3‐year DM: Arm A: 17.3%, Arm B: 16.9%	3 years
El Husseini et al. Retrospective unicentric, 2021 (El Husseini et al. 2021) [18]	81 TNT *n* = 26, CRT *n* = 55	TNT: 38.5%, CRT: 27.3%	NR	TNT: 42.3%, CRT: 35.8%	NR	NR	CRT: 54.5% received any no of AdjCT cycles, 40% received a full course of CT TNT: 100% received neoadjuvant CT, 84.6% received a full course (*p* < 0.01)	NR	2‐year DFS: 81% vs. 84% (*p* = 0.15)	NR	TNT: 22.7 months CRT: 47.8 months
PRODIGE‐23, Phase III RCT, 2021 (Conroy et al. 2021) [6]	461 ICT *n* = 231, CRT *n* = 230	ICT: 27.8%, RCT: 12.1% (*p* < 0.0001)	ICT: 95.3%, CRT:94.4% (*p* = 0.625)	NR	NR	Grade 3–4 toxicity during adjuvant CT ICT: 45.1%, CRT: 74.1% (*p* < 0.0001), Grade 3–4 toxicity during induction CT in ICT arm 46·5%, Grade 3–4 toxicity during CRT: similar in both groups (*p* = 0.883)	Received FOLFIRINOX in ICT arm: 97·8%, Received complete 6 cycles of FOLFIRINOX: 91·6%, Received ≥ 80% of planned doses: 72·6%, Received planned CRT ICT: 94·8%, CRT: 98·7% (*p* = 0·019) Received AdjCT: ICT: 77·3%, CRT: 78·6% (*p* = 0·749) Received ≥ 80% of AdjCT planned doses (first 3 months): ICT:21·9%, CRT:50·6% (*p* < 0·0001)	3‐year OS ICT: 90.8%, CRT: 87.7% (*p* = 0.077)	3‐year DFS ICT: 75.7%, CRT: 68.5% (*p* = 0.034)	Postop metastatic progression: ICT: 1%, CRT:4.7% (*p* = 0.030) 3‐year MFS: ICT: 78.8%, CRT:71.1% (*p* = 0.017), Locoregional relapse: ICT: 4.3%, CRT:0.5.7% (*p* = 0.51)	46.5 months

Abbreviations: cCR, clinical complete response; CR, complete response; CRT, chemoradiotherapy; CT, chemotherapy; DFS, disease‐free survival; DM, distant metastasis; ICT, induction chemotherapy; LAR, low anterior resection; LR, local recurrence; MFS, metastasis‐free survivalNOM, nonoperative management; NR, not reported; OS, overall survival; pCR, pathological complete response; RCT, randomized clinical trial; RT, radiotherapy; TNT, total neoadjuvant therapy.

### 2.2 Consolidation Chemotherapy Studies

In contrast to induction chemotherapy, some investigators suggested a TNT approach that starts with radiation and is followed by chemotherapy. The idea behind this approach is that consolidation chemotherapy can be more effective in achieving pCR if there is a longer interval between radiation therapy and surgery [19]. Previously, it has been shown that delayed surgery within 1–2 months after RT improves tumor downstaging [20]. Furthermore, radiation therapy is more potent than chemotherapy when it comes to treating local symptoms, and it can provide early and long‐lasting palliation [21]. Through an effective control of local symptoms by upfront RT, patients can better tolerate the chemotherapy course that follows [22]. Another rationale behind administering Consolidation chemotherapy is the possibility of the development of radio‐resistant clones after receiving neoadjuvant chemotherapy in nonresponder cells, as chemotherapy may interfere with the apoptosis process, reducing the sensitivity of tumor cells to subsequent radiation therapy. Additionally, chemotherapy may cause EGFR overexpression in some tumor cells, resulting in increased angiogenesis and stromal proliferation, leading to radioresistance. It is possible that prompt treatment response may not guarantee favorable long‐term results [23, 24]. Now, we review studies that used consolidation chemotherapy as a proposed TNT regimen.

A Phase 2, multicentric, nonrandomized, open‐label trial by Garcia‐Aguilar et al. was conducted to assess the effectiveness and safety of various treatment methods in individuals with Stage II or III rectal adenocarcinoma, specifically those with T3–4, N0 or any T, N1–2. The study comprised four treatment groups, each receiving fluorouracil‐based chemoradiotherapy followed by different cycles of mFOLFOX6 chemotherapy and surgery. Group 1 received fluorouracil‐based concurrent chemoradiotherapy (CCRT) followed by surgery. Group 2 underwent fluorouracil‐based chemoradiotherapy (CRT), followed by two cycles of mFOLFOX6 chemotherapy before surgery. Group 3 followed a similar regimen to Group 2 but received four cycles of mFOLFOX6 chemotherapy before surgery. Group 4 received the same CRT as group 2 but underwent a more intensive chemotherapy regimen, with six cycles of mFOLFOX6 before surgery. The study observed that the pathological complete response rate rose notably with each study group, reaching 38% in group 4 from 18% in Group 1. A comparison between Group 4 and the standard regimen showed a significant association with pCR. In further analyses, treatment intensity measured by mFOLFOX6 cycles and the chemoradiation‐to‐surgery interval also proved significant predictors of PCR. Overall, Grade 3 adverse events during chemoradiation were reported in 26% of patients, with no difference among study groups. Consolidation chemotherapy (mFOLFOX6) led to an increase in adverse events from 3% to 28%. However, the percentage of patients who had sphincter preservation surgery and negative margin reports after the procedure did not show a significant difference across the groups. Pelvic fibrosis increased dramatically in Groups 2 to 4, but the technical difficulty of the surgery remained consistent [25]. After a median follow‐up of 59 months, the final results showed that DFS was meaningfully linked with the study group, ypTNM stage, and pCR [26].

In the Polish II trial in 2016, investigators conducted a phase III RCT to compare the effectiveness of CRT followed by surgery to that of short‐course radiotherapy (SCRT) 5 × 5 Gy plus three cycles of consolidation chemotherapy with FOLFOX4 after RT compared to long‐course CRT followed by surgery in 515 eligible patients with T3 and T4 rectal cancer. The patients were divided into two arms: The SCRT arm (261 patients) received 25 Gy in 5 fractions followed by FOLFOX4, and the CRT arm (254 patients) underwent long‐course RT consisting of 50.4 Gy with concurrent oxaliplatin,5‐FU, and leucovorin. The study found that R0 resection rates were slightly higher in SCRT than in CRT (77% vs. 71%, *p* = 0.07). pCR rates were 16% vs. 12% in SCRT and CRT arms, respectively (*p* = 0.17). The 3‐year overall survival was higher in SCRT than in the CRT arm (73% vs. 65%, *p* = 0.046). The 8‐year overall survival was the same in both groups, at 49%. Additionally, the 8‐year disease‐free survival rate was 43% for patients who received consolidation chemotherapy, compared with 41% for the standard CRT group (*p* = 0.6). Notably, patients in the SCRT arm experienced significantly lower preoperative acute toxicity, while late complications and sphincter preservation rates were similar between the groups. The findings suggest that while both treatments are effective, short‐course radiotherapy with consolidation chemotherapy offers advantages in overall survival and lower toxicity [27, 28].

Zhai and colleagues performed a retrospective analysis in 2021 to assess the safety and efficacy of consolidation chemotherapy in 197 individuals diagnosed with LARC. The study examined the outcomes of two sets of patients. Pelvic intensity‐modulated radiation therapy (IMRT) along with two rounds of CAPOX (capecitabine and oxaliplatin) followed by surgery was administered to the first group consisting of 81 patients. The second group, known as the total neoadjuvant therapy (TNT) group, received three cycles of CAPOX after chemoradiation, followed by surgery. The TNT group showed a significantly higher rate of pCR of 32.7% vs. 12.8% in the standard treatment group (*p* = 0.002). Both groups did not report any serious adverse effects or deaths during CRT or consolidation chemotherapy [29].

The RAPIDO Phase III trial, led by Bahadoer, compared the effectiveness of a TNT protocol, which included SCRT and consolidation chemotherapy afterward, with the standard treatment of long‐course chemoradiotherapy in treating high‐risk LARC. High‐risk LARC was defined as the presence of any of the following factors: T4, N2, extramural venous invasion (EMVI), lateral lymph node involvement, and mesorectal fascia (MRF) involvement. The trial involved 912 patients, out of which 450 received the standard CRT with TME, and 462 underwent SCRT followed by consolidation chemotherapy (FOLFOX or XELOX) and then surgery. The study observed that the TNT group had a significantly higher pCR rate at 28.4% compared with 14.3% in the standard CRT group. Additionally, the experimental group had a lower 3‐year disease‐related treatment failure rate of 23.7% in comparison with 30.4% in the standard treatment group (*p* = 0.019). However, there was no significant difference in 3‐year OS between the two groups. Adverse events of Grade 3 or higher occurred in 48% of patients in the experimental group. In comparison, only 25% of patients who received standard‐of‐care treatment had such adverse events. The most common Grade 3 adverse event experienced during preoperative treatment was diarrhea, which affected both groups but was more common in the experimental group. Overall, Serious adverse events occurred in 38% of experimental group patients and 34% in the standard‐of‐care group patients, regardless of receiving adjuvant chemotherapy [7]. After a median follow‐up time of 5.6 years, the locoregional recurrence rate was higher in the TNT group (10% vs. 6%, *p* = 0.027), while the locoregional failure rate did not meet the significance level. Additionally, the cumulative probability of disease‐related treatment failure and distant metastasis was reported to be significantly lower in the TNT group than in the standard CRT group (27.8% vs. 34%, *p* = 0.048, and 23% vs. 30.4%, *p* = 0.011, respectively). Although the cumulative probability of OS was comparable between the two arms [30].

In a clinical trial called WAIT, 49 patients who had rectal cancer were divided into two groups. The first group underwent a standard 10‐week wait after receiving chemoradiotherapy before surgery (SCRT group, 24 patients). In contrast, the other group received an additional three cycles of chemotherapy with fluorouracil during the same wait period after chemoradiotherapy (XCR group, 25 patients). The two groups did not demonstrate a notable difference in the rate of pCR, as concluded by the study. pCR occurred in six patients in the SCRT group and four patients in the XCR group (*p* = 0.49) [31].

The PREPARE (KSCG CO 10–03) trial enrolled 110 T3‐4M0 rectal cancer patients. The study compared two treatment options: the CRT arm, which involved chemoradiotherapy (50.4 Gy in 28 fractions) with capecitabine followed by TME, and the consolidation chemotherapy (CC) arm, which involved two cycles of CAPOX after chemoradiotherapy and before TME. The results showed that 21.2% of patients in CRT and 36.4% in the CC arm experienced downstaging, although the difference was not statistically significant (*p* = 0.077). The pCR rate was 5.8% vs. 13.6% in CRT and CC arms, respectively (*p* = 0.167). Adverse events of grade ≥ 3 during preoperative treatment occurred in 3.6% vs. 9.4% of patients in CRT and CC arms, respectively. During postoperative recovery, Grade 3 AE occurred in 1.9% vs. 9.0% of patients, respectively [32].

The phase III STELLAR trial examined patients with T3‐4 primary tumor or regional lymph node‐positive rectal cancer. They were randomly assigned to one of two groups. The first group, called the TNT group, included 302 patients who received short‐course radiotherapy (25 Gy over 1 week) followed by four cycles of consolidation chemotherapy and then TME, followed by two additional cycles of postoperative CAPOX. The second group, called the CRT group, consisted of 297 patients who underwent long‐course CRT (50 Gy over 5 weeks with concurrent capecitabine), followed by TME and six cycles of adjuvant CAPOX. After 35 months of follow‐up, the researchers discovered that both groups had similar 3‐year DFS rates. The DFS rate in the TNT group was non‐inferior to the CRT group (64.5% vs. 62.3%; HR = 0.883, *p* < 0.001 for noninferiority). However, the TNT group had a higher 3‐year overall survival rate than the CRT group (86.5% vs. 75.1%; *p* = 0.033). There were no significant differences in metastasis‐free survival or locoregional recurrence between the two groups. The researchers also found that acute Grade 3–4 toxicities during preoperative treatment were significantly more common in the TNT group than in the CRT group (26.5% vs. 12.6%; *p* < 0.001) [33]. It's important to note that adjuvant chemotherapy is not typically part of the TNT protocol. In this study, however, both groups received adjuvant therapy, which helped maintain some level of consistency in comparisons. However, the number of chemotherapy cycles differed between the groups: the CRT group received all 6 cycles of CAPOX as adjuvant chemotherapy, while the TNT group received 4 cycles as consolidation chemotherapy and only 2 cycles as adjuvant therapy. Interestingly, among patients who received adjuvant chemotherapy, a higher percentage in the TNT group completed the planned cycles compared with the CRT group (60% vs. 48.3%; *p* = 0.009). Furthermore, the incidence of Grade 3 or higher toxicities with adjuvant chemotherapy was significantly lower in the TNT group compared to the CRT group (3.3% vs. 11.8%; *p* = 0.003). This suggests that despite the additional adjuvant therapy cycles in the CRT group, patients in the TNT group tolerated the adjuvant chemotherapy better, with fewer severe toxicities.

The ENSEMBLE‐1 Phase II trial was carried out at five institutions in Japan and was open‐label and single‐arm. Patients had to meet specific criteria, including being at least 20 years old and having resectable nonmetastatic LARC with a clinical stage of T3‐4N0 or TanyN+M0 at the time of diagnosis. The treatment protocol consisted of neoadjuvant short‐course radiation therapy (SCRT) 5 Gy for 5 days with a total dose of 25 Gy plus six cycles of CAPOX, followed by TME. If a clinical complete response (cCR) was achieved during the preoperative assessment, nonoperative management (NOM) was permitted. Finally, the primary endpoint among 30 included patients was the pathological complete response rate. The completion rates were 100% for SCRT and 83% for CAPOX. TME and NOM were performed on twenty and seven patients, respectively. pCR was observed in six patients (30% [95% CI 14.0%–50.8%]). The primary endpoint was met. pCR + cCR was observed in 43.3% of patients. There were no treatment‐related deaths. Grade 3 or higher adverse events in more than 20% of patients were diarrhea (23.3%) and neutropenia (23.3%). The median follow‐up period was 15.6 months, with no recurrence or regrowth in NOM. The rate of pCR is promising in this study and is considered higher than that of many induction CT‐based TNT studies [34].

**FIGURE 2:**
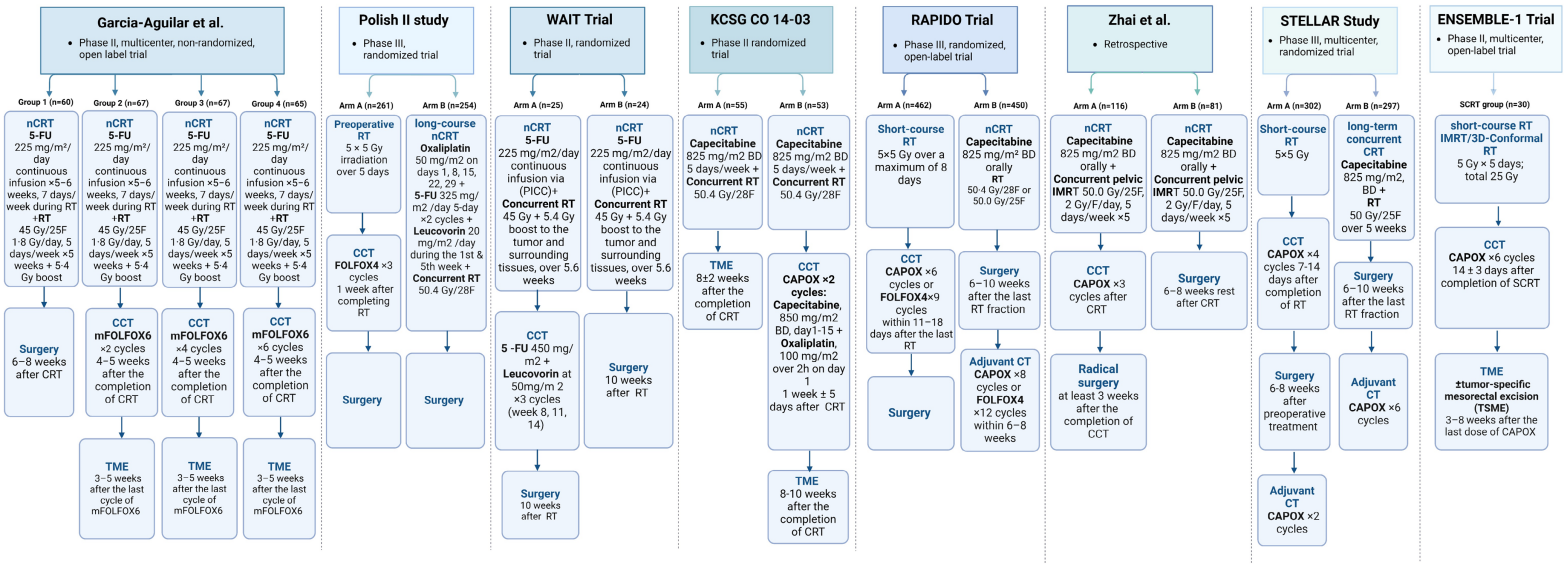
Design of studies evaluating consolidation chemotherapy as a TNT approach. This figure illustrates the design of eight studies evaluating the use of consolidation chemotherapy within a total neoadjuvant therapy approach for locally advanced rectal cancer. It compares various regimens, including neoadjuvant chemoradiotherapy, short‐course or long‐course radiotherapy followed by consolidation chemotherapy, and the timing of surgery. Each study represents a different approach to integrating consolidation chemotherapy into the TNT framework to improve treatment outcomes. CAPOX, Capecitabine, 1000 mg oral BD Days 1–14 + oxaliplatin 130 mg/m2 Day 1, Q3 weeks; CCT, consolidation chemotherapy; CT, chemotherapy; FOLFOX4, Oxaliplatin 85 mg/m^2^ IV day 1 + Leucovorin 200 mg/m^2^ IV days 1,2 + FU 400 mg/m^2^ IV + FU 600 mg/m^2^ IV for 22 h days 1,2.IMRT, intensity‐modulated radiation therapy; mFOLFOX6, Oxaliplatin 85 mg/m^2^ 2h IV + Leucovorin 400 mg/m^2^ 2h IV + Fluorouracil 400 mg/m^2^ IV then 2400 mg/m^2^ CIV over 46 h Q2 weeks; nCRT, neoadjuvant chemoradiotherapy; NOM, nonoperative management; RT, radiation therapy; TME, total mesorectal excision.

Consolidation chemotherapy has been shown to improve pathological response, particularly in low‐lying tumors where a “watch and wait” approach may be considered. It is also beneficial to start treatment with radiotherapy for symptomatic patients requiring a rapid local response. Table 2 summarizes the relevant articles, and Figure 2 illustrates the study designs.

**TABLE 2:** Results of studies evaluating consolidative chemotherapy.

Study	Number of participants	PCR	R0 resection	Downstaging	Sphincter preservation surgery	Toxicity	Treatment completion rate per protocol	OS	DFS	LR/DM	Median f/u
Garcia‐Aguilar et al. Phase II, nonrandomized trial, 2015 (Garcia‐Aguilar et al. 2015; Marco et al. 2018) [25, 26]	Group 1: *n* = 60, Group 2: *n* = 67, Group 3: *n* = 67, Group 4: *n* = 65	Group 1:18%, Group 2: 25%, Group 3: 30%, Group 4: 38% (*p* = 0.0036)	Group1: 98%, Group 2: 100%, Group 3: 96%, Group 4: 100% (*p* = 0.089)	NR	Group 1: 77%, Group 2: 75%, Group 3: 75%, Group 4: 68% (*p* = 0.68)	CRTGrade 3 AE: 26%, no difference in study groups, FOLFOX grade 3–4 AEs: Group 2: 4%, Group 3: 18%, Group 4: 36%	Proportion of patients completing all planned cycles of mFOLFOX6: Group 2: 82%, Group 3: 81%, Group 4: 77% (*p* = 0·50)	Group 1: 79% Group 2: 92% Group 3: 88% Group 4: 84% (*p* = 0.37)	Group 1: 50%, Group 2: 81%, Group 3: 86% Group 4: 76% (*p* = 0.004)	NR	59 months
POLISH II, Phase III RCT, 2016 (Cisel et al. 2019; Bujko et al. 2016) [27, 28]	515 Arm A (Short‐course RT + CCT) *n* = 261 Arm B (CRT) *n* = 254	Arm A: 16%, Arm B: 12% (*p* = 0.17)	Arm A: 77%, Arm B: 71% (*p* = 0.07)	NR	Anterior resection: Arm A: 50%, Arm B: 49%	Toxicity of any grade Arm A: 75%, Arm B: 83% (*p* = 0.006) Grade 3/4 AEs: Arm A: 23%, Arm B: 21%, Toxic deaths: Arm A: 1%, Arm B: 3%	RT dose reduction (< 5 × 5 or < 50 Gy): Arm A: 0%, Arm B: 8% (*p* < 0.001), RT and/or CT dose reduction and/or delays due to toxicity: Arm A: 37%, Arm B: 34% (*p* = 0.40), Oxaliplatin administration: Arm A:72%, Arm B: 64% (*p* = 0.062)	8‐year OS 49% in both arms (*p* = 0.38) Median OS: Arm A:89 months, Arm B: 81 months	8‐year DFS Arm A: 43%, Arm B: 41% (*p* = 0.65)	DM: Arm A: 36%, Arm B:34% (*p* = 0.54) LR: Arm A: 35%, Arm B: 32% (*p* = 0.60)	84 months
WAIT, Phase II RCT, 2017 (Moore et al. 2017) [31]	49 SCRT *n* = 24, XCRT *n* = 25	SCRT:25%, XCRT:16% (*p* = 0.49) cCR: SCRT: 8.3%, XCRT: 12% (*p* = 1.0)	SCRT: 91.7%, XCRT: 92% (*p* = 0.50)	NR	Anterior resection: SCRT: 50%, XCRT: 72% (*p* = 0.19)	No deaths attributable to either CRT or CT during the wait period	NR	NR	NR	NR	NR
PREPARE (KCSG CO 14–03), Phase II RCT, 2018 (Kim et al. 2018) [33]	108 CRT *n* = 55, CCT *n* = 53	CCT:13.6%, CRT:5.8% *p* = 0.167	CCT: 88.6%, CRT: 100%	CCT:36.4%, CRT:21.2% (*p* = 0.077)	Sphincter‐saving surgery CCT: 93.2%, CRT: 100%	Grade ≥ 3 AEs preoperative: CCT:9.4%, CRT:3.6% Grade ≥ 3 postoperative AEs: CCT:9%, CRT: 1.9%	NR	NR	NR	NR	NR
RAPIDO, Phase III RCT, 2021 (Bahadoer et al. 2021; Dijkstra et al. 2023) [7]	912 CCT *n* = 462, CRT *n* = 450	CCT: 28.4%, CRT: 14.3% (*p* < 0.0001)	CCT: 90.3%, CRT: 90.4% (*p* = 0.87)	NR	NR	Grade ≥ 3 AEs during preoperative tx: 48% vs. 25% Grade ≥ 3 AEs during Adj tx:34% of standard‐of‐care tx	Completed preoperative CT: CCT: 85%, CRT: 90%, Stopping CT due to toxicity: CCT:14%, CRT: 7% during preoperative tx, CRT: 32% during adjuvant tx	3‐year OS: CCT: 89.1%, CRT: 88.8% (*p* = 0.59)	NR	LR: CCT: 5%, CRT: 3% DM rate: CCT: 67%, CRT: 81% 3‐year LRF cumulative probability: CCT: 8.3%, CRT: 6% (*p* = 0·12) 3‐year DM cumulative probability: CCT: 20%, CRT: 26.8% (*p* = 0.0048) F/U After 5.6 years: LRF: CCT:11.7%, CRT: 8.1% (*p* = 0.07), LRR: CCT: 10.2%, CRT: 6.1% (*p* = 0.027)	5.6 years
Zhai et al. retrospective, 2021 (Zhai et al. 2021) [29]	TNT CRT	TNT: 32.7%, CRT: 12.8% (*p* = 0.002)	NR	NR	LAR: TNT: 65.5%, CRT: 77%	Grade 3 acute AEs during neoadjuvant tx: Neutropenia in 7.4% CRT group and 10.3% in TNT group, Thrombocytopenia 4.9% in CRT group and 7.8% in TNT group, Diarrhea in 3.7% CRT group and 6.7% in TNT group, Rectal pain in 4.9% CRT group and 6% in TNT group, Grade 3 anemia and radiation dermatitis 3.7% in CRT group and 5.2% in TNT group	Received all 3 cycles of CAPOX in TNT group: 95.7% 4.3% of patients in the TNT group received two cycles of CAPOX due to grade 3 neutropenia	NR	NR	NR	NR
STELLAR, Phase III RCT, 2022 (Jin et al. 2022) [33]	599 TNT *n* = 302 CRT *n* = 297	pCR + cCR: TNT: 21.8%, CRT: 12.3% (*p* = 0.002) pCR: TNT: 71.1%, CRT:68.7% (*p* = 0.578)	TNT: 91.5%, CRT: 87.8% (*p* = 0.189)	NR	Anterior resection: TNT: 47.2%, CRT: 52.6%	Grade 3–4 toxicities during preoperative tx: TNT:26.5%, CRT: 12.6% (*p* < 0.001) Grade 3–4 toxicities during Adj tx: TNT:3.3%, CRT:11.8% (*p* = 0.003)	Completion rate of preoperative tx: TNT: 82.6, CRT: 95.2% (*p* < 0.001) full‐dose completion rate of preoperative tx: TNT: 74.8%, CRT:93.2% (*p* < 0.001)	3‐year OS TNT: 86.5%, CRT: 75.1% (*p* = 0.033)	3‐year DFS TNT:64.5%CRT: 62.3% (HR = 0.88, 95%CI: not applicable to 1.11; Noninferiority test *p* < 0.001)	DM rate: TNT: 21.5%, CRT: 22.6% LRR rate: TNT:6.6%, CRT: 7.7%, 3‐year MFS: TNT: 77.1%, CRT: 75.3% (*p* = 0.475) 3‐year LRR: TNT: 8.4, CRT: 11% (*p* = 0.461)	35 months
ENSEMBLE‐1, Phase II trial, 2023 (Kagawa et al. 2023) [34]	30	pCR: 30% cCR: 43%	100%	T‐downstaging: 70%, N‐downstaging: 50%	LAR+ISR: 90%	No treatment‐related deaths CAPOX‐related Grade ≥ 3AEs (CTCAE ver. 5.0) (≥ 20%), including diarrhea (23.3%) and neutropenia (23.3%).	Completion of Short‐course RT: 100%, Completion of CAPOX: 83.3%, Completion of TNT: 83.3%	NR	NR	No recurrence or regrowth in NOM	15.6 months

Abbreviations: AE, adverse event; cCR, clinical complete response; CCT, consolidation chemotherapy; CRT, chemoradiotherapy; CT, chemotherapy; DFS, disease‐free survival; DM, distant metastasis; ISR, intersphincteric resection; LAR, low anterior resection; LR, local recurrence; LRF, locoregional failure; LRR, locoregional recurrence; MFS, metastasis‐free survival; NR, not reported; OS, overall survival; pCR, pathological complete response; RCT, randomized clinical trial; RT, radiotherapy; SCRT, standard chemoradiotherapy; TNT, total neoadjuvant therapy; XCRT, extended chemoradiotherapy.

### 2.3 Comparative Studies of Induction vs. Consolidation

Two key studies, the CAO/ARO/AIO‐12 and the OPRA trial, have significantly influenced the optimal sequencing of TNT for LARC, albeit with different endpoints. By analyzing and integrating the findings of these studies within the broader context of evolving treatment strategies, we can better understand how to tailor treatment sequences for improved patient outcomes. These studies have provided crucial insights, incrementally advancing our understanding of the optimal sequence of treatment modalities.

In the CAO/ARO/AIO‐12 study, led by Fokas et al. the authors compared the pathological complete response (pCR) rates and toxicity between induction chemotherapy followed by chemoradiotherapy (IC‐CRT) versus CRT followed by consolidation chemotherapy (CRT‐CC). The winner should have improved the hypothesized 15% pCR rate of standard CRT to 25%. The finding of a higher pCR rate in CRT‐CC than in the IC‐CRT arm (25% vs. 17%, *p* < 0.001 and *p* = 0.201 compared to the hypothesis) underscores the potential of post‐CRT chemotherapy to improve tumor responsiveness. Consolidation chemotherapy enhances patient tolerance to chemoradiation. Also, Grade 3–4 adverse events were less common in the CRT‐CC arm than in the induction chemotherapy protocol. After a 43‐month follow‐up, the 3‐year DFS, LRR, and DM and also the quality of life were not different between the two schedules, and the delay of chemotherapy did not deteriorate the systemic spread of the disease [35, 36]. The investigators claimed that, due to higher pCR, upfront CRT and consolidation chemotherapy is the prior approach if the goal is organ preservation.

Building upon these insights, the OPRA trial, orchestrated by Gracia Aguilar and the team, expands the discourse by comparing the DFS and OS outcomes of induction chemotherapy followed by CRT (IC‐CRT) and CRT followed by consolidation chemotherapy (CRT‐CC). Both ICT‐CRT and CRT‐CCT groups had a 3‐year DFS rate of 76%, consistent with the historical 3‐year DFS rate of 75%. The 3‐year TME‐free survival rates were 41% and 53% in the INCT‐CRT and CRT‐CNCT groups, respectively. The groups did not show any significant difference in terms of survival measures. The investigation found comparable DFS rates in patients who underwent TME after restaging and those who received TME after regrowth. Long‐term follow‐up (5.1 years) maintained comparable 5‐year DFS rates (71% vs. 69%). However, TME‐free survival favored the CRT‐CNCT group (54% vs. 39%, *p* = 0.012). Most regrowth occurred within 2 years, with DFS similar regardless of TME timing. While DFS and OS were found to be identical between the groups, a novel observation was the higher inclination toward a “watch and wait” approach in the CRT‐CNCT arm [37, 38]. This outcome introduces a nuanced perspective on patient management, suggesting that the sequence of treatments not only impacts tumor response but may also influence treatment strategies, including the consideration of nonsurgical management in responsive cases.

The juxtaposition of these studies illuminates a critical progression in our understanding of TNT sequencing. The CAO/ARO/AIO‐12 study establishes a benchmark for the potential of consolidation chemotherapy to enhance pCR rates and improve tolerability. Concurrently, the OPRA trial extends this knowledge by examining broader treatment outcomes and patient management strategies, suggesting that the benefits of consolidation chemotherapy may also include greater flexibility in adopting less invasive management options.

Collectively, these studies contribute to a more nuanced understanding of TNT sequencing. They advocate for a tailored approach where the choice between induction and consolidation chemotherapy is informed by individual patient characteristics, tumor response, and the overarching goal of optimizing both survival outcomes and quality of life. The emerging consensus suggests that while consolidation chemotherapy may be preferable for patients requiring symptom relief and potentially higher pCR rates, induction chemotherapy remains a valuable strategy for addressing micrometastasis early in the treatment course.

In 2023, Moyer et al. conducted a multicenter retrospective study involving patients diagnosed with clinical Stage II–III rectal cancer. During the study, patients at the University of Colorado (2016–2020) underwent induction chemotherapy followed by long‐course CRT, while those at Washington University (2017–2020) received short‐course radiotherapy followed by consolidative chemotherapy. Of the 84 patients who received induction chemotherapy and chemoradiation and the 83 who received short‐course radiotherapy and consolidative chemotherapy, clinical complete response rates were similar among those who underwent complete restaging evaluation (49% vs. 53%, *p* = 0.659). In the induction chemotherapy and chemoradiation group, 80% proceeded to surgery, with 28% achieving a complete pathological response. Meanwhile, in the short‐course radiotherapy and consolidative chemotherapy group, 53% had surgery, with 11% achieving pCR. In general, a complete response was observed in 43% of patients in the IC group, compared with 53% who received short‐course radiotherapy and CCT (*p* = 0.189). Perioperative outcomes were similar between the two groups regarding intraoperative complications (2% vs. 7%), complete mesorectal specimen (85% vs. 84%), anastomotic leak (9% vs. 7%), organ or space infection (9% vs. 5%), readmission (19% vs. 21%), and reoperation (8% vs. 9%) (all *p* > 0.05). The study concluded that in patients with Clinical Stage II or III rectal cancer, the TNT approach, whether upfront chemotherapy followed by chemoradiation or short‐course radiotherapy followed by consolidative chemotherapy, resulted in similar perioperative morbidity and complete response rates [39].

**FIGURE 3:**
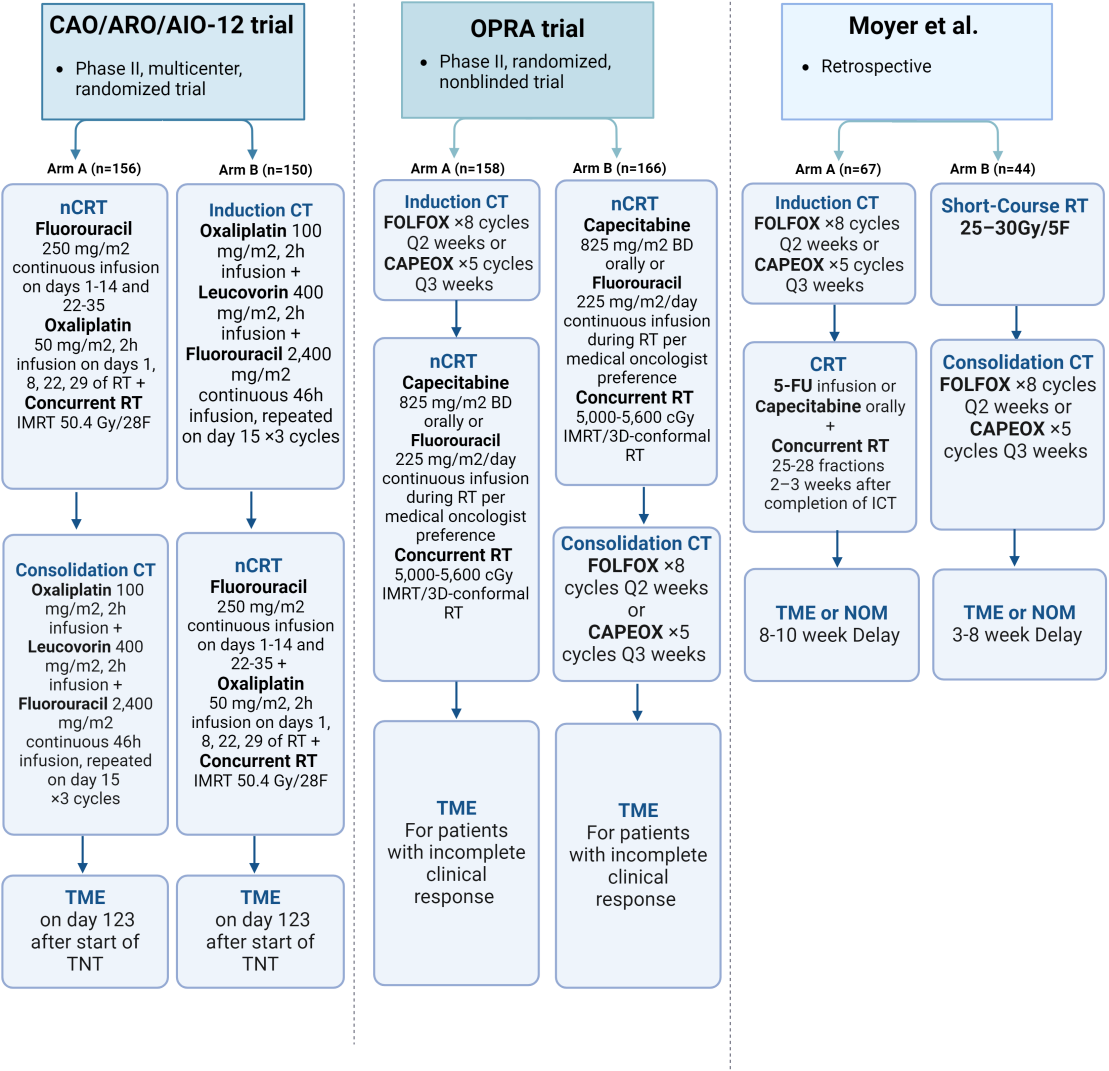
Design of studies comparing CCT‐TNT and ICT‐TNT approach. This figure summarizes studies comparing the consolidation chemotherapy versus the induction chemotherapy as part of the TNT approach in locally advanced rectal cancer treatment. The studies include the CAO/ARO/AIO‐12 trial (Phase II, multicenter, randomized), the OPRA trial (Phase II, randomized, nonblinded), and a retrospective study by Moyer et al. Each study is depicted with two arms, detailing the sequence of induction chemotherapy, neoadjuvant chemoradiotherapy, consolidation chemotherapy, and the timing for total mesorectal excision or nonoperative management. CAPEOX, Capecitabine, 1000 mg oral BD Days 1–14 + oxaliplatin 130 mg/m2 Day 1, Q3 weeks.CT, chemotherapy; FOLFOX, Oxaliplatin 85 mg/m2 + 5‐FU CIV infusion 2400 mg/m2 Days 1–3 ± 5‐FU 400 mg/m2 IV bolus + Leucovorin 400 mg/m2 Q2 weeks; nCRT, neoadjuvant chemoradiotherapy; NOM, nonoperative management; RT, radiation therapy; TME, total mesorectal excision.

As the landscape of TNT for locally advanced rectal cancer evolves, these studies underscore the importance of ongoing research and clinical trials. They highlight the need for a dynamic and adaptable treatment paradigm that considers the latest evidence to optimize treatment sequences for the best possible patient outcomes. A visual representation of the study designs can be found in Figure 3, and Table 3 summarizes the findings.

**TABLE 3:** Results of studies comparing induction and consolidation chemotherapy.

Study	Number of patients	PCR	R0 resection	Downstaging	Sphincter preservation	Toxicity	Treatment completion per protocol	OS	DFS	LR/DM	Median f/u
CAO/ARO/AIO‐12, 2019, Phase II RCT + long‐term results	306 Arm A (ICT) *n* = 156, Arm B (CCT) *n* = 150	ICT:17%, CRT: 25% pCR + cCR: ICT:21%, CRT: 28%	ICT: 92%, CRT: 90%	NR	ICT: 68%, CRT: 72%	ICT: Grade 3–4 CRT‐related AEs: 37% CT‐related AEs:22% CRT: grade 3–4 CRT‐related AEs:27% CT‐related AEs:22%	Received full‐dose RT: ICT: 91%, CCT: 97% Received full‐dose FU during CRT: ICT: 78%, CCT:87% Received full dose of Oxaliplatin during CRT: ICT: 76%, CCT: 93% Received all cycles of Oxaliplatin in induction/consolidation: ICT:92%, CCT:85% Received all cycles of FU in induction/consolidation CT: ICT: 93%, CCT:90%	3‐year OS 92% in both groups (*p* = 0.81)	3‐year DFS 73% in both groups (*p* = 0.82)	3‐year cumulative incidence LR: ICT: 6%, CRT: 5% (*p* = 0.67), 3‐year DM cumulative incidence: ICT: 18%, CRT: 16% (*p* = 0.52)	43 months
OPRA, 2022, Phase II RCT	324 ICT‐CRT *n* = 158 CRT‐CCT *n* = 166	ypT0: ICT: 8%, CCT: 9%, ypN0: ICT: 85%, CCT: 73%	ICT: 91%, CCT: 88%	NR	Preserved rectum at 3 years in ITT: ICT: 41%, CCT: 53% (*p* = 0.01) TME‐free survival: ICT: 47%, CCT: 60% (*p* = 0.02) Transanal Excision: ICT:5%, CCT:6%	Grade ≥ 3 AEs during TNT: ICT: 41%, CCT: 34%	Received FOLFOX: ICT: 74%, CCT:70% Received eight cycles of FOLFOX: ICT:86%, CCT:84% Received CAPEOX: ICT: 21%, CCT: 20% Received five cycles of CAPEOX: ICT:85%, CCT:88% Started RT: ICT: 93%, CCT:98%, Median radiation dose: ICT: 5400 (5040‐5400) Gy, CCT: 5400 (5040‐5600) Gy Received concurrent FU or capecitabine: ICT: 98%, CCT: 100%	NR	3‐year DFS: 76% in both groups, no significant difference with the historical group (75%)	DMFS: ICT: 84%, CCT: 82% LRFS: 94% in both groups	3 years
Moyer et al. 2023, [39] Retrospective Multicenter	167 ICT *n* = 84, CCT *n* = 83	cCR: ICT:49%, CCT: 53% (*p* = 0.659) pCR: ICT: 28.4%, CCT: 11.4% (*p* = 0.033) CR: ICT:43%, CCT:53% (*p* = 0.189)	NR	NR	ICT: 73%, CCT: 71% (*p* = 0.758)	Intraoperative complication: 2% vs. 7% Complete mesorectal specimen: 85% vs. 84% anastomotic leak (9% vs. 7%), organ/space infection (9% vs. 5%), readmission (19% vs. 21%), reoperation (8% vs. 9%), (all *p* > 0.05).	completion of CT: ICT:91%, CCT:80% (*p* = 0.083), Receive IMRT: ICT: 78%, CCT: 30% Receive 3D‐CRT: ICT:15%, CCT:70% (*p* < 0.001)	NR	NR	NR	NR

Abbreviations: AE, adverse event; cCR, clinical complete response; CCT, consolidation chemotherapy; CR, complete response; CRT, chemoradiotherapy; CT, chemotherapy; DFS, disease‐free survival; DM, distant metastasis; DMFS, distant metastasis‐free survival; ICT, induction chemotherapy; IMRT, intensity‐modulated radiation therapy; ITT, intention‐t‐ treat; LR, local recurrence; LRFS, locoregional‐free survival; NR, not reported; OS, overall survival; pCR, pathological complete response; RCT, randomized clinical trial; RT, radiotherapy; TME, total mesorectal excision; TNT, total neoadjuvant therapy.

This refined analysis not only encapsulates the findings of the two pivotal studies but also situates them within the larger trajectory of treatment optimization for locally advanced rectal cancer, demonstrating how each piece of evidence contributes to a layered understanding of optimal treatment sequencing.

### 2.4 Future Directions

Exploring novel treatment strategies and optimizing multimodal treatment approaches to improve TNT outcomes and the patient's quality of life and to lower the risk of local regrowth or distant metastasis through an organ‐preserving strategy is crucial. In recent years, targeted therapies or immunotherapy, including anti‐PD1 or anti‐PD‐L1 agents, have emerged as promising new combinational treatment options with total neoadjuvant therapy during chemotherapy, radiotherapy, or CRT. For instance, a Phase III trial compares the safety profile, pCR rate, and DFS rates between total neoadjuvant treatment with or without immunotherapy in high‐risk locally advanced rectal cancer (NCT06229041).

Another approach involves combining immunotherapy with standard CRT or chemotherapy regimens. For example, a trial investigates the efficacy of Nivolumab, an anti‐PD‐1, plus FOLFOX regimen as consolidation therapy after CRT (NCT03921684). A Phase II/III trial (STELLAR II Study) explores how well neoadjuvant short‐course RT and chemotherapy with or without PD‐1 inhibitors followed by TME or watch‐and‐wait strategy works in patients with locally advanced rectal adenocarcinoma (NCT05484024).

Emerging evidence on integrating PD‐1/PD‐L1 inhibitors with nCRT in mismatch repair proficient (pMMR)/microsatellite stable (MSS) LARC patients introduces an innovative dimension to the treatment landscape. Small‐scale studies indicate that this combination could yield better short‐term outcomes compared to historical data. Nonetheless, introducing these immunotherapeutic agents amidst unresolved questions regarding the ideal sequencing and the optimal dose and fractionation of radiotherapy in this combined regimen presents considerable challenges [40]. This underscores the imperative need for further research and prolonged follow‐up to elucidate the chronic complications and long‐term clinical outcomes associated with these advanced treatment modalities.

In a single‐arm Phase II study, patients will receive durvalumab, an anti‐PD‐L1, every 4 weeks throughout the entire process, including induction chemotherapy, CRT, and the rest period before surgery. Researchers theorized that starting durvalumab at the first chemotherapy session and maintaining it throughout the 6‐week CRT phase may enhance its effectiveness according to the neoantigen‐inducing effects of platinum‐based chemotherapy and chemoradiotherapy (NCT04293419).

An ongoing trial (NCT04926324) is evaluating the survival metrics of adding different doses of targeted therapies, including PARP inhibitors combined with an anti‐PD‐1 agent, to radiotherapy. Another ongoing trial (NCT05763927) is assessing the pathological outcomes and survival rates of administering induction therapy using toripalimab, an anti‐PD‐1 agent, and fruquintinib, an anti‐VEGF agent, followed by short‐course RT and surgery.

Moreover, researchers are exploring novel approaches to decrease treatment‐related toxicities and improve patient quality of life. A recent clinical trial is evaluating the effect of adding chlorophyllin, sodium copper complex, to neoadjuvant chemoradiation therapy on gastrointestinal toxicity and quality of life (NCT05856305). It is also essential for future trials of the TNT approach to assess patient‐reported outcomes and quality‐of‐life measures. Long‐term follow‐up studies are also needed to evaluate complications and survival rates.

In addition to exploring new treatment strategies, providing precise assessment tools, including imaging or molecular techniques, during the follow‐up period as predictors of relapse is vital. Utilizing artificial intelligence methods and machine learning, it is possible to examine various types of biological data over a period of time. This approach offers the opportunity to predict which patients would derive optimal benefits from both total neoadjuvant therapy (TNT) and a watch‐and‐wait strategy [41].

In rectal cancer management within the TNT approach, advanced molecular technologies like the SignateraTM assay have been used in an observational study (NCT05356585) to analyze circulating tumor DNA fragments in blood derived from rectal tumor biopsies. This technique helps clinicians gain real‐time insights into tumor biology and treatment response and minimizes disease recurrence.

Accurately identifying high‐risk imaging features is important in the context of total neoadjuvant therapy for rectal cancer. Results of a preliminary analysis of a study highlight the use of DW‐MRI as a potential predictive tool of response to treatment and could further help detect the most probable subjects for total neoadjuvant therapy in terms of achieving pCR [42]. In this context, radiomics, that is, extraction and analysis of quantitative parameters from radiological imaging, is proving to be a promising noninvasive method in the prediction of tumor response and long‐term outcomes and valuable for incorporation into future studies [43, 44].

## 3 Conclusions

This narrative review provided a comprehensive exploration of total neoadjuvant therapy (TNT) in the management of locally advanced rectal cancer (LARC), emphasizing the critical evaluation of treatment sequencing. Our analysis highlights that TNT, through its induction and consolidation chemotherapy strategies, significantly advances the therapeutic paradigm, enhancing pathological response rates, disease‐free survival, and potentially overall survival, all while maintaining a manageable profile of adverse events.

When considering the sequence of RT, it is important to note the differences between major studies investigating the TNT approach. These include the chemotherapy regimen and cycles, the time interval between RT and surgery, the risk profiles of participants, the RT dose and technique, the surgical protocols, and response evaluations. Additionally, many studies lack detailed reporting on downstaging and organ preservation/sphincter‐sparing rates. While percentages of TN stages before and after treatment are provided, the absence of specific downstaging analysis limits a thorough assessment of the overall pathological response among all stages. This underscores a significant research gap and highlights the need for more consistent and detailed reporting of these critical outcomes in future studies.

The nuanced comparison between induction and consolidation chemotherapy within TNT reveals distinct benefits. Induction chemotherapy is particularly effective in early intervention against micrometastasis, particularly in patients susceptible to the spread of disease such as middle or upper T2‐3a/b and N2 disease or those with MRI‐defined T2‐3a/b and extra‐mural venous invasion. However, this may lead to decreased subsequent chemoradiotherapy adherence and selection of resistant clones. On the other hand, consolidation chemotherapy has been associated with higher pCR rates and improved patient tolerability, suggesting its suitability for patients requiring symptom relief, those at risk of developing local recurrence such as MRI‐defined T3c/d‐4 or a positive circumferential radial margin or those eligible for a nonoperative management by “watch and wait” approach particularly in low‐lying tumors.

Now, the biggest challenge ahead is the lack of a reliable trial that can provide an answer to the question, “What is the most effective sequence of RT in TNT protocols?” The only trial available is the German Phase II CAO/ARO/AIO‐12, which mainly focuses on pCR as an endpoint. However, this endpoint has become outdated and replaced by newer endpoints such as combined complete response or major response. These new endpoints pave the way for nonoperative management and watch‐and‐wait strategies. OPRA trial, another Phase II study, has used these modern endpoints and has yielded better results in terms of surgery‐free survival for those who began with RT and continued with consolidation chemotherapy. Thus, the most up‐to‐date study supports the approach that includes upfront long‐course CRT and consolidation chemotherapy, even though it is not yet a Phase III trial.

Tailoring chemotherapy and incorporating immunotherapeutic agents for the treatment of locally advanced rectal cancer is essential to maximize treatment efficacy and improve patient outcomes. Personalized medicine and predictive biomarkers will play a key role in refining treatment strategies. Ongoing clinical trials and studies will pave the way toward effectively managing this challenging condition.

## Author Contributions

**Reza Ghalehtaki:** conceptualization (lead), investigation (equal), methodology (equal), project administration (lead), resources (equal), supervision (lead), validation (lead), visualization (equal), writing – original draft (equal), writing – review and editing (equal). **Forouzan Nourbakhsh:** investigation (equal), methodology (equal), resources (equal), validation (equal), writing – original draft (equal), writing – review and editing (equal). **Romina Abyaneh:** investigation (equal), methodology (equal), resources (equal), validation (equal), visualization (equal), writing – review and editing (equal). **Azadeh Sharifian:** data curation (equal), investigation (equal), resources (equal), writing – original draft (equal). **Sheyda Pashapour‐Khoyi:** investigation (equal), resources (equal), writing – original draft (equal). **Mahdi Aghili:** resources (equal), supervision (equal), validation (equal), writing – review and editing (equal). **Maria Antonietta Gambacorta:** supervision (equal), validation (equal), writing – review and editing (equal). **Felipe Couñago:** supervision (equal), validation (equal), writing – review and editing (equal).

## Ethics Statement

The authors have nothing to report.

## Consent

The authors have nothing to report.

## Conflicts of Interest

The authors declare no conflicts of interest.

## Data Availability

The authors have nothing to report.

## References

[1] M. Aghili , N. Khalili , N. Khalili , et al., “Short‐Course Versus Long‐Course Neoadjuvant Chemoradiotherapy in Patients With Rectal Cancer: Preliminary Results of a Randomized Controlled Trial,” Radiation Oncology Journal 38, no. 2 (2020): 119–128. 33012155 10.3857/roj.2020.00115 PMC7533412

[2] R. Okamura , Y. Itatani , Y. Fujita , et al., “Postoperative Recurrence in Locally Advanced Rectal Cancer: How Does Neoadjuvant Treatment Affect Recurrence Pattern?,” World Journal of Surgical Oncology 21, no. 1 (2023): 247. 37587422 10.1186/s12957-023-03136-0 PMC10428603

[3] F. Sclafani , C. Corro , and T. Koessler , “Debating Pros and Cons of Total Neoadjuvant Therapy in Rectal Cancer,” Cancers (Basel) 13, no. 24 (2021): 6361. 34944980 10.3390/cancers13246361 PMC8699289

[4] F. Petrelli , F. Trevisan , M. Cabiddu , et al., “Total Neoadjuvant Therapy in Rectal Cancer: A Systematic Review and Meta‐Analysis of Treatment Outcomes,” Annals of Surgery 271, no. 3 (2020): 440–448. 31318794 10.1097/SLA.0000000000003471

[5] M. C. Riesco‐Martinez , C. Fernandez‐Martos , C. Gravalos‐Castro , et al., “Impact of Total Neoadjuvant Therapy vs. Standard Chemoradiotherapy in Locally Advanced Rectal Cancer: A Systematic Review and Meta‐Analysis of Randomized Trials,” Cancers (Basel) 12, no. 12 (2020): 3655. 33291454 10.3390/cancers12123655 PMC7762140

[6] T. Conroy , J. F. Bosset , P. L. Etienne , et al., “Neoadjuvant Chemotherapy With FOLFIRINOX and Preoperative Chemoradiotherapy for Patients With Locally Advanced Rectal Cancer (UNICANCER‐PRODIGE 23): A Multicentre, Randomised, Open‐Label, Phase 3 Trial,” Lancet Oncology 22, no. 5 (2021): 702–715. 33862000 10.1016/S1470-2045(21)00079-6

[7] R. R. Bahadoer , E. A. Dijkstra , B. van Etten , et al., “Short‐Course Radiotherapy Followed by Chemotherapy Before Total Mesorectal Excision (TME) Versus Preoperative Chemoradiotherapy, TME, and Optional Adjuvant Chemotherapy in Locally Advanced Rectal Cancer (RAPIDO): A Randomised, Open‐Label, Phase 3 Trial,” Lancet Oncology 22, no. 1 (2021): 29–42. 33301740 10.1016/S1470-2045(20)30555-6

[8] A. B. H. Bhatti , A. Waheed , A. Hafeez , et al., “Can Induction Chemotherapy Before Concurrent Chemoradiation Impact Circumferential Resection Margin Positivity and Survival in Low Rectal Cancers?,” Asian Pacific Journal of Cancer Prevention 16, no. 7 (2015): 2993–2998. 25854395 10.7314/apjcp.2015.16.7.2993

[9] S. Feng , P. Yan , Q. Zhang , et al., “Induction Chemotherapy Followed by Neoadjuvant Chemoradiotherapy and Surgery for Patients With Locally Advanced Rectal Cancer: A Systematic Review and Meta‐Analysis,” International Journal of Colorectal Disease 35 (2020): 1355–1369. 32488419 10.1007/s00384-020-03621-y

[10] S. Y. Ng , K. L. Colborn , L. Cambridge , et al., “Induction Chemotherapy Reduces Patient‐Reported Toxicities During Neoadjuvant Chemoradiation With Intensity Modulated Radiotherapy for Rectal Cancer,” Clinical Colorectal Cancer 18, no. 3 (2019): 167–174. 31104990 10.1016/j.clcc.2019.04.001 PMC6817304

[11] C. Fernandez‐Martos , C. Pericay , J. Aparicio , et al., “Phase II, Randomized Study of Concomitant Chemoradiotherapy Followed by Surgery and Adjuvant Capecitabine Plus Oxaliplatin (CAPOX) Compared With Induction CAPOX Followed by Concomitant Chemoradiotherapy and Surgery in Magnetic Resonance Imaging‐Defined, Locally Advanced Rectal Cancer: Grupo Cancer de Recto 3 Study,” Journal of Clinical Oncology 28, no. 5 (2010): 859–865. 20065174 10.1200/JCO.2009.25.8541

[12] C. Fernandez‐Martos , X. Garcia‐Albeniz , C. Pericay , et al., “Chemoradiation, Surgery and Adjuvant Chemotherapy Versus Induction Chemotherapy Followed by Chemoradiation and Surgery: Long‐Term Results of the Spanish GCR‐3 Phase II Randomized Trialdagger,” Annals of Oncology 26, no. 8 (2015): 1722–1728. 25957330 10.1093/annonc/mdv223

[13] M. L. Conces and A. Mahipal , “Adoption of Total Neoadjuvant Therapy in the Treatment of Locally Advanced Rectal Cancer,” Current Oncology 31, no. 1 (2024): 366–382. 38248109 10.3390/curroncol31010024 PMC10813931

[14] R. Marechal , B. Vos , M. Polus , et al., “Short Course Chemotherapy Followed by Concomitant Chemoradiotherapy and Surgery in Locally Advanced Rectal Cancer: A Randomized Multicentric Phase II Study,” Annals of Oncology 23, no. 6 (2012): 1525–1530. 22039087 10.1093/annonc/mdr473

[15] A. Cercek , C. S. D. Roxburgh , P. Strombom , et al., “Adoption of Total Neoadjuvant Therapy for Locally Advanced Rectal Cancer,” JAMA Oncology 4, no. 6 (2018): e180071. 29566109 10.1001/jamaoncol.2018.0071 PMC5885165

[16] C. Fernández‐Martos , C. Pericay , F. Losa , et al., “Effect of Aflibercept Plus Modified FOLFOX6 Induction Chemotherapy Before Standard Chemoradiotherapy and Surgery in Patients With High‐Risk Rectal Adenocarcinoma: The GEMCAD 1402 Randomized Clinical Trial,” JAMA Oncology 5, no. 11 (2019): 1566–1573. 31465088 10.1001/jamaoncol.2019.2294 PMC6865228

[17] D. Pesántez , S. Ten Hoorn , I. Machado , et al., “Total Neoadjuvant Therapy With or Without Aflibercept in Rectal Cancer: 3‐Year Results of GEMCAD‐1402,” JNCI Journal of the National Cancer Institute 115, no. 12 (2023): 1497–1505. 37405857 10.1093/jnci/djad120

[18] Z. El Husseini , Y. Haibe , Y. Bouferraa , et al., “Total Neoadjuvant Therapy in Patients With Locally Advanced Rectal Cancer: A Tertiary Medical Center Experience,” Molecular and Clinical Oncology 15, no. 4 (2021): 1–6. 10.3892/mco.2021.2382 PMC8408679 34476104

[19] C.‐K. Liao , Y.‐T. Kuo , Y.‐J. Hsu , et al., “Effect of Short‐Course Radiotherapy Followed by Oxaliplatin‐Based Consolidation Chemotherapy on Organ Preservation in Locally Advanced Rectal Cancer,” International Journal of Colorectal Disease 38, no. 1 (2023): 92. 37022513 10.1007/s00384-023-04388-8

[20] D. Pettersson , E. Lörinc , T. Holm , et al., “Tumour Regression in the Randomized Stockholm III Trial of Radiotherapy Regimens for Rectal Cancer,” Journal of British Surgery 102, no. 8 (2015): 972–978. 10.1002/bjs.9811 PMC4744683 26095256

[21] A. S. Myint and J. P. Gérard , “Role of Radiotherapy in the Treatment of Rectal Cancer in Older Patients,” European Journal of Surgical Oncology 46, no. 3 (2020): 349–357. 31926607 10.1016/j.ejso.2019.12.017

[22] H. Zhang , G. Li , K. Cao , et al., “Impact of Total Neoadjuvant Therapy Consisting of Consolidation Chemotherapy on Locally Advanced Rectal Cancer Survival,” International Journal of Colorectal Disease 37, no. 7 (2022): 1657–1668. 35716183 10.1007/s00384-022-04179-7

[23] T. De Pas , G. Pelosi , F. de Braud , et al., “Modulation of Epidermal Growth Factor Receptor Status by Chemotherapy in Patients With Locally Advanced Non–Small‐Cell Lung Cancer Is Rare,” Journal of Clinical Oncology 22, no. 24 (2004): 4966–4970. 15611511 10.1200/JCO.2004.01.195

[24] R. Glynne‐Jones , J. Grainger , M. Harrison , P. Ostler , and A. Makris , “Neoadjuvant Chemotherapy Prior to Preoperative Chemoradiation or Radiation in Rectal Cancer: Should We Be More Cautious?,” British Journal of Cancer 94, no. 3 (2006): 363–371. 16465172 10.1038/sj.bjc.6602960 PMC2361136

[25] J. Garcia‐Aguilar , O. S. Chow , D. D. Smith , et al., “Effect of Adding mFOLFOX6 After Neoadjuvant Chemoradiation in Locally Advanced Rectal Cancer: A Multicentre, Phase 2 Trial,” Lancet Oncology 16, no. 8 (2015): 957–966. 26187751 10.1016/S1470-2045(15)00004-2 PMC4670237

[26] M. R. Marco , L. Zhou , S. Patil , et al., “Consolidation mFOLFOX6 Chemotherapy After Chemoradiotherapy Improves Survival in Patients With Locally Advanced Rectal Cancer: Final Results of a Multicenter Phase II Trial,” Diseases of the Colon & Rectum 61, no. 10 (2018): 1146–1155. 30192323 10.1097/DCR.0000000000001207 PMC6130918

[27] B. Cisel , L. Pietrzak , W. Michalski , et al., “Long‐Course Preoperative Chemoradiation Versus 5 × 5 Gy and Consolidation Chemotherapy for Clinical T4 and Fixed Clinical T3 Rectal Cancer: Long‐Term Results of the Randomized Polish II Study,” Annals of Oncology 30, no. 8 (2019): 1298–1303. 31192355 10.1093/annonc/mdz186

[28] K. Bujko , L. Wyrwicz , A. Rutkowski , et al., “Long‐Course Oxaliplatin‐Based Preoperative Chemoradiation Versus 5× 5 Gy and Consolidation Chemotherapy for cT4 or Fixed cT3 Rectal Cancer: Results of a Randomized Phase III Study,” Annals of Oncology 27, no. 5 (2016): 834–842. 26884592 10.1093/annonc/mdw062

[29] Z. Zhai , K. Zhang , C. Wang , et al., “Adding Three Cycles of CAPOX After Neoadjuvant Chemoradiotherapy Increases the Rates of Complete Response for Locally Advanced Rectal Cancer,” Current Oncology 28, no. 1 (2021): 283–293. 33419188 10.3390/curroncol28010033 PMC7903282

[30] E. A. Dijkstra , P. J. Nilsson , G. A. Hospers , et al., “Locoregional Failure During and After Short‐Course Radiotherapy Followed by Chemotherapy and Surgery Compared With Long‐Course Chemoradiotherapy and Surgery: A 5‐Year Follow‐Up of the RAPIDO Trial,” Annals of Surgery 278, no. 4 (2023): e766–e772. 36661037 10.1097/SLA.0000000000005799 PMC10481913

[31] J. Moore , T. Price , S. Carruthers , et al., “Prospective Randomized Trial of Neoadjuvant Chemotherapy During the ‘wait period’ Following Preoperative Chemoradiotherapy for Rectal Cancer: Results of the WAIT Trial,” Colorectal Disease 19, no. 11 (2017): 973–979. 28503826 10.1111/codi.13724

[32] S. Y. Kim , J. Joo , T. W. Kim , et al., “A Randomized Phase 2 Trial of Consolidation Chemotherapy After Preoperative Chemoradiation Therapy Versus Chemoradiation Therapy Alone for Locally Advanced Rectal Cancer: KCSG CO 14‐03,” International Journal of Radiation Oncology, Biology, Physics 101, no. 4 (2018): 889–899. 29976501 10.1016/j.ijrobp.2018.04.013

[33] J. Jin , Y. Tang , C. Hu , et al., “Multicenter, Randomized, Phase III Trial of Short‐Term Radiotherapy Plus Chemotherapy Versus Long‐Term Chemoradiotherapy in Locally Advanced Rectal Cancer (STELLAR),” Journal of Clinical Oncology 40, no. 15 (2022): 1681–1692. 35263150 10.1200/JCO.21.01667 PMC9113208

[34] Y. Kagawa , J. Watanabe , M. Uemura , et al., “Short‐Term Outcomes of a Prospective Multicenter Phase II Trial of Total Neoadjuvant Therapy for Locally Advanced Rectal Cancer in Japan (ENSEMBLE‐1),” Annals of Gastroenterological Surgery 7, no. 6 (2023): 968–976. 37927927 10.1002/ags3.12715 PMC10623965

[35] E. Fokas , M. Allgauer , B. Polat , et al., “Randomized Phase II Trial of Chemoradiotherapy Plus Induction or Consolidation Chemotherapy as Total Neoadjuvant Therapy for Locally Advanced Rectal Cancer: CAO/ARO/AIO‐12,” Journal of Clinical Oncology 37, no. 34 (2019): 3212–3222. 31150315 10.1200/JCO.19.00308

[36] E. Fokas , A. Schlenska‐Lange , B. Polat , et al., “Chemoradiotherapy Plus Induction or Consolidation Chemotherapy as Total Neoadjuvant Therapy for Patients With Locally Advanced Rectal Cancer: Long‐Term Results of the CAO/ARO/AIO‐12 Randomized Clinical Trial,” JAMA Oncology 8, no. 1 (2022): e215445. 34792531 10.1001/jamaoncol.2021.5445 PMC8603234

[37] J. Garcia‐Aguilar , S. Patil , M. J. Gollub , et al., “Organ Preservation in Patients With Rectal Adenocarcinoma Treated With Total Neoadjuvant Therapy,” Journal of Clinical Oncology 40, no. 23 (2022): 2546–2556. 35483010 10.1200/JCO.22.00032 PMC9362876

[38] F. S. Verheij , D. M. Omer , H. Williams , et al., “Long‐Term Results of Organ Preservation in Patients With Rectal Adenocarcinoma Treated With Total Neoadjuvant Therapy: The Randomized Phase II OPRA Trial,” Journal of Clinical Oncology 42, no. 5 (2024): 500–506. 37883738 10.1200/JCO.23.01208 PMC11578087

[39] A. M. Moyer , J. D. Vogel , S. H. Lai , et al., “Total Neoadjuvant Therapy in Rectal Cancer: Multi‐Center Comparison of Induction Chemotherapy and Long‐Course Chemoradiation Versus Short‐Course Radiation and Consolidative Chemotherapy,” Journal of Gastrointestinal Surgery 27, no. 5 (2023): 980–989. 36759387 10.1007/s11605-023-05601-3

[40] K. X. Lin , A. C. Istl , D. Quan , A. Skaro , E. Tang , and X. Zheng , “PD‐1 and PD‐L1 Inhibitors in Cold Colorectal Cancer: Challenges and Strategies,” Cancer Immunology, Immunotherapy 72, no. 12 (2023): 3875–3893. 37831146 10.1007/s00262-023-03520-5 PMC10700246

[41] Y. Kagawa , J. J. Smith , E. Fokas , et al., “Future Direction of Total Neoadjuvant Therapy for Locally Advanced Rectal Cancer. Nature Reviews,” Gastroenterology & Hepatology 21 (2024): 1–12. 38485756 10.1038/s41575-024-00900-9 PMC11588332

[42] F. Iafrate , F. Ciccarelli , G. M. Masci , et al., “Predictive Role of Diffusion‐Weighted MRI in the Assessment of Response to Total Neoadjuvant Therapy in Locally Advanced Rectal Cancer,” European Radiology 33, no. 2 (2023): 854–862. 35980431 10.1007/s00330-022-09086-7

[43] G. Chiloiro , P. Rodriguez‐Carnero , J. Lenkowicz , et al., “Delta Radiomics Can Predict Distant Metastasis in Locally Advanced Rectal Cancer: The Challenge to Personalize the Cure,” Frontiers in Oncology 10 (2020): 595012. 33344243 10.3389/fonc.2020.595012 PMC7744725

[44] N. Dinapoli , B. Barbaro , R. Gatta , et al., “Magnetic Resonance, Vendor‐Independent, Intensity Histogram Analysis Predicting Pathologic Complete Response After Radiochemotherapy of Rectal Cancer. International Journal of Radiation Oncology* Biology*,” Physics 102, no. 4 (2018): 765–774. 10.1016/j.ijrobp.2018.04.065 29891200

